# Does compulsive behavior in Anorexia Nervosa resemble an addiction? A qualitative investigation

**DOI:** 10.3389/fpsyg.2015.01608

**Published:** 2015-10-20

**Authors:** Lauren R. Godier, Rebecca J. Park

**Affiliations:** Oxford Brain-Body Research into Eating Disorders, Department of Psychiatry, University of OxfordOxford, UK

**Keywords:** Anorexia Nervosa, eating disorders, compulsivity, qualitative, addictions

## Abstract

The characteristic relentless self-starvation behavior seen in Anorexia Nervosa (AN) has been described as evidence of compulsivity, with increasing suggestion of parallels with addictive behavior. This study used a thematic qualitative analysis to investigate the parallels between compulsive behavior in AN and Substance Use Disorders (SUD). Forty individuals currently suffering from AN completed an online questionnaire reflecting on their experience of compulsive behavior in AN. Eight main themes emerged from thematic qualitative analysis; compulsivity as central to AN, impaired control, escalating compulsions, emotional triggers, negative reactions, detrimental continuation of behavior, functional impairment, and role in recovery. These results suggested that individuals with AN view the compulsive nature of their behavior as central to the maintenance of their disorder, and as a significant barrier to recovery. The themes that emerged also showed parallels with the DSM-V criteria for SUDs, mapping onto the four groups of criteria (impaired control, social impairment, risky use of substance, pharmacological criteria). These results emphasize the need for further research to explore the possible parallels in behavioral and neural underpinnings of compulsivity in AN and SUDs, which may inform novel treatment avenues for AN.

## Introduction

Anorexia nervosa (AN) is a severely debilitating psychiatric disorder characterized by an intense fear of weight gain or becoming fat, despite significantly low body weight (American Psychiatric Association, 2013). Individuals with AN place extreme over-importance on the control of weight and shape, and often have disturbed body image perception (Fairburn et al., 2003). These distorted beliefs and perceptions are accompanied by a perpetual drive for thinness, and continuous lowering of weight goals (Barbarich-Marsteller et al., 2011). The characteristic behaviors seen in AN to achieve weight loss goals, such as extreme dietary restriction and over-exercise, have been described as evidence of the compulsive nature of the disorder (Godier and Park, 2014a; Park et al., 2014). Individuals with AN may develop compulsive, ritualized behaviors in an attempt to neutralize the anxiety associated with persistent, intrusive thoughts regarding food and weight gain (Steinglass and Walsh, 2006).

Compulsivity is defined as a trait in which actions are persistently repeated despite adverse consequences (Robbins et al., 2012). This can be seen in the drug-seeking behavior characteristic of substance dependence, in which a lack of control is felt over behavior, despite the adverse consequences of drug taking (Kalivas and Volkow, 2005). A comparison between the compulsive nature of behavior in AN and addiction has been increasingly suggested (Scheurink et al., 2010; Zink and Weinberger, 2010; Barbarich-Marsteller et al., 2011; Kaye et al., 2013; Godier and Park, 2014a; Park et al., 2014). The developmental period of onset is similar in both disorders, with an initial phase of reward seeking, in the form of weight loss in AN, which is experienced as rewarding and pleasurable (Scheurink et al., 2010; Park et al., 2011, 2012), as if it were a drug. Furthermore, individuals find it increasingly difficult refraining from weight loss behavior despite adverse consequences (Park et al., 2011, 2012). Behavioral and neurobiological similarities are also evident between the two disorders (Godier and Park, 2014a). Both disorders are associated with deficits in tasks that tap cognitive flexibility (Holliday et al., 2005; Saraswat et al., 2006; Steinglass et al., 2006; Sarrar et al., 2011; Bühren et al., 2012; Izquierdo and Jentsch, 2012; Hildebrandt et al., 2015), defined as a rigid cognitive style, which is suggested to contribute to compulsivity (Fineberg et al., 2010). Furthermore, dysfunction in the cortico-striatal circuitry suggested to underpin compulsivity (Robbins, 2007; Fineberg et al., 2010), can also be seen in AN and substance dependence (Everitt and Robbins, 2005; Rothemund et al., 2011; Godier and Park, 2014a).

The commonalities in compulsive behaviors across disorders has been highlighted by Robbins et al. (2012), who propose a transdiagnostic approach to compulsivity, arguing that this behavioral construct has cross-diagnostic significance. They posit that compulsive behavior across diagnoses may be underpinned by similar behavioral and neurobiological mechanisms, which manifest in a disorder-specific manner. A transdiagnostic approach to compulsivity may aid in the development of novel treatment avenues relating to specific behaviors, rather than focusing on diagnosis (Robbins et al., 2012).

Despite increasing interest in the transdiagnostic relevance of compulsive behavior across psychiatric disorders (Robbins et al., 2012; Godier and Park, 2014a), and recent suggestions implicating compulsive and habitual weight loss behaviors in the persistence of AN (Walsh, 2013), there is a paucity of research directly investigating parallels in the compulsive behavior seen in AN and substance dependence. However, our group recently found that adapting the criteria for addiction was a useful way of capturing compulsive self-starvation behavior in AN (Godier and Park, 2014b). In this study, we adapted the questions of the Yale Food Addiction Scale (YFAS) (Gearhardt et al., 2009), a questionnaire developed using the DSM-IV criteria for substance dependence, for self-starvation behavior (the Self-Starvation Scale, SS). This measure was successfully validated in a community and clinical AN sample (Godier and Park, 2014b). The utility of using these criteria in capturing self-starvation behavior, supports the conceptualization of compulsivity as a transdiagnostic construct of behavior, and highlights the importance of further research investigating the compulsive nature of weight loss behavior in AN.

A better understanding of the behavioral parallels between AN and substance dependence may guide further research investigating the underlying mechanisms of behavior in AN, and ultimately aid the development of novel treatment strategies targeting these behaviors. With this in mind, this study aimed to examine the validity of the suggested parallels between AN and substance addiction with a qualitative investigation of the subjective experience of compulsive weight loss behavior in AN. Participants in this study were asked to reflect on their experience of compulsive behavior in AN, and its potential role in the persistence of the disorder. The themes that emerged from the qualitative analysis were compared to the DSM-V criteria for substance use disorders (SUD), with the hypothesis that overlap in the qualitative themes and these criteria would be observed.

## Methods

### Participants

Participants between the ages of 18–65 were recruited for a group of individuals currently suffering from AN (*n* = 40). Participants were recruited via email, internet, and poster advertisement. This included advertisement on the BEAT website. Additionally, a number of participants were recruited from the Oxford Research List for Anorexia Nervosa (ORLA) of individuals with experience of AN, maintained by the research team at the University of Oxford. Participants in this study were recruited as part of a larger questionnaire study carried out by our research group (See Godier and Park, 2014b). The study was described to potential participants as an investigation of two new measures of compulsive behavior in AN, and the concept of compulsive behavior in AN was described to participants in the information sheet. Participants were recruited on the basis of a self-reported diagnosis of AN from a healthcare professional, and a self-reported current BMI of below 18.5.

### Study procedure

The research protocol was reviewed and approved by the NRES Committee South Central—Berkshire B (Ref: 13/SC/1370). Data was collected using the Bristol Online Survey system (BOS; Institute of Learning and Research Technology, University of Bristol, UK). Participants were initially sent an information sheet and asked some screening questions via email to ensure eligibility for the study. Participants were then provided a link to an online consent form and their responses to a number of questions were recorded online. Participants completed the Eating Disorder-Questionnaire (EDE-Q; Fairburn and Beglin, 2008) and Clinical Impairment Assessment (CIA; Bohn and Fairburn, 2008) described below, to measure eating disorder symptoms, followed by the compulsivity and demographic questions.

### Measures

Participants completed online questionnaire measures of ED symptoms (EDE-Q; Fairburn and Beglin, 2008) and clinical impairment (CIA; Bohn and Fairburn, 2008). Participants were asked to reflect on their experience of compulsive behavior in AN, and their responses were recorded online. There was no space or time limitation and participants could write as much or as little as they felt was appropriate. Participants read the following information and questions as prompts for their responses:

We are interested in any comments or reflections you may have about the compulsive and driven nature of behavior in anorexia (e.g., excessive exercise and food restriction) and your experience of this.

Please fill in the box below with any comments you have on the following:

1. What is your experience of compulsion in anorexia- how does it feel?
2. How troubled do you feel by compulsive behaviors—and what is most troubling?
3. How important do you think the compulsive nature of behavior is the course of anorexia—its development and/or recovery?
4. How do you feel when you are prevented from engaging in any compulsive behavior?
5. Does anything help reduce your sense of compulsion, and/ or the compulsive behaviors that trouble you?
6. Any other comments.

Participants also provided the following demographic information:

- Age
- Age of onset of Anorexia Nervosa
- Duration of eating disorder
- Type(s) of treatment received (e.g., inpatient, outpatient, day patient)
- Lowest BMI (or height and weight if unknown)
- Current BMI (or height and weight if unknown)

### Qualitative analysis

Responses were downloaded from BOS and analyzed using Thematic Analysis (TA) techniques (Braun and Clarke, 2006). A deductive coding method was employed, guided by previous theory and the hypotheses of the current study, whilst allowing for novel themes to arise from the data. Coding and analysis was carried out by hand.

Based on the guidelines for using TA outlined by Braun and Clarke (2006), transcripts were initially read and reread a number of times to facilitate familiarization with the data set. Initial codes were generated manually by hand by noting key words/phrases of interest in the margin of the transcripts, and the entire data set was systematically coded. These initial codes were compared and sorted into themes, grouping similar codes together under one broader theme. The content of the themes was reviewed in order to develop appropriate titles. A thematic map was then created in order to visualize and explore the relationships between themes. The relationships between themes were reviewed, and themes were refined into a number of main and sub themes. Finally, the thematic map was reviewed in relation to the entire data set, and themes were further refined in order to best reflect the data as a whole.

To ensure the inter-rater reliability of the coding system, the transcripts were coded again by a second researcher, using the final coding scheme. This independent researcher was a post-doctoral research assistant in the research team who had experience of working with EDs, but was unfamiliar with the specific hypotheses of the current study. The independent researcher coded 10 of the 40 excerpts, and inter-rater reliability was assessed by comparing the codes given by the independent reader to the original coded data set. This resulted in 89% agreement between researchers in application of the final coding scheme. The independent researcher felt the final coding scheme was an accurate reflection of the data; however, discussion of discrepancies in coding between researchers led to further revision of the final data coding. Following this inter-rater reliability check, the final interpretation of the data and conclusions drawn were discussed with the research team.

**Figure 1:**
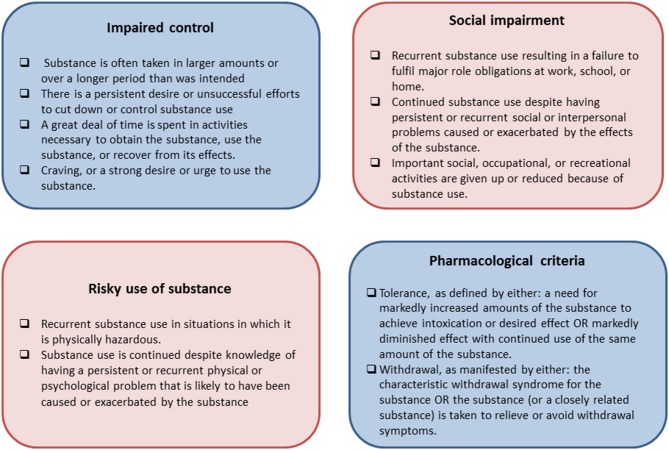
**The four groups of the DSM-V diagnostic criteria for Substance Use Disorders**.

**Figure 2:**
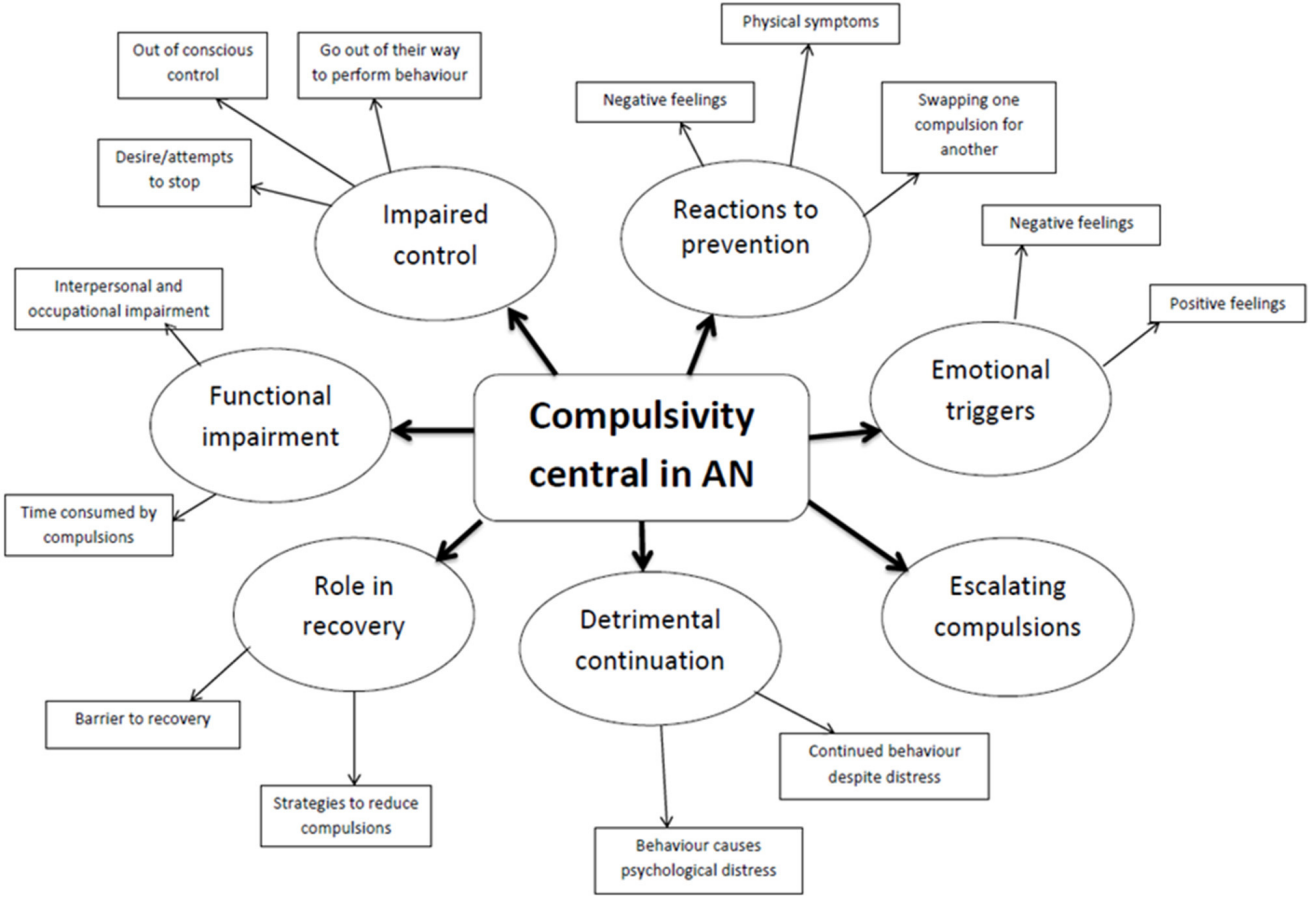
**Thematic map of the themes and subthemes extracted from the data**.

The final themes were then assessed for parallels with the DSM-V diagnostic criteria for SUDs (See Figure 1) in line with the predictions of the study. These criteria are grouped into four themes as shown in Figure 2, assessing impaired control, social impairment, risky use of the substance, and pharmalogical criteria.

## Results

### Demographic results

Forty participants reporting a current diagnosis of AN completed the online questionnaire. Of these 40 participants 39 were female and 1 was male. Twenty-two of the participants had received inpatient treatment at least once, 12 had received day patient treatment, and 35 had received treatment as an outpatient. Table 1 shows scores on ED psychopathology (EDE-Q and CIA), average age, age of onset of AN, length of AN, average current BMI, and average lowest BMI for all participants. Fifteen participants reported binge and/or purge symptoms in the past 28 days (4 reported binge and purge symptoms, 3 reported binge symptoms only, 8 reported purge symptoms only).

**Table 1:** **Demographic information (***N*** = 40)**.

	**Mean**	**Range**
EDE-Q (global)	4.06	1.09–5.85
CIA	33.35	7–48
Age	28.0	18–60
Age of Onset	16.5	13–27
Length of AN (years)	11.5	1–43
Current BMI	15.9	13.3–18.5
Lowest BMI	13.4	10–15.8

*EDE-Q, Eating Disorder Examination Questionnaire; CIA, Clinical Impairment Assessment; BMI, Body Mass Index*.

### Qualitative results

The average number of words per participant was 192, although the variance between participants was great (minimum: 24, maximum: 1405). Using the final coding system resulted in 78.8% of the data set being coded. Eight main themes were identified, within which 14 sub-themes were included, as shown as a thematic map in Figure 2, and in Table 2. The endorsement rate for each main and sub theme is also shown in Table 2. Each theme and sub-theme is presented below with verbatim excerpts.

**Table 2:** **Participant endorsement rates of the main and sub-themes extracted from the thematic qualitative analysis**.

**Theme**	**Sub-themes**	**Endorsement rates**
1. Compulsivity is central to AN		15/40 (37.5%)
2. Impaired control		25/40 (62.5%)
	2.1 Out of conscious control	25/40 (62.5%)
	2.2 Go out of their way to perform behavior	5/40 (12.5%)
	2.3 Desire/attempts to stop	12/40 (30%)
3. Functional impairment		15/40 (37.5%)
	3.1 Time consumed by compulsions	8/40 (20%)
	3.2 Interpersonal and occupational impairment	11/40 (27.5%)
4. Detrimental continuation		18/40 (45%)
	4.1 Continued behavior despite distress	11/40 (27.5%)
	4.2 Behavior causes psychological distress	16/40 (40%)
5. Escalating compulsions		14/40 (35%)
6. Reactions to prevention		29/40 (72.5%)
	6.1 Negative feelings	29/40 (72.5%)
	6.2 Physical feelings	9/40 (22.5%)
	6.3 Swapping one compulsion for another	5/40 (12.5%)
7. Emotional triggers		16/40 (40%)
	7.1 Negative feelings	13/40 (32.5%)
	7.2 Positive feelings	7/40 (17.5%)
8. Role in recovery		26/40 (65%)
	8.1 Compulsivity as a barrier to recovery	21/40 (52.5%)
	8.2 Strategies to reduce compulsions	20/40 (50%)

#### Theme one: Compulsivity is central to AN

As the prompt questions in this study lead participants to base their answers around the compulsive nature of behavior in AN, and due to the deductive nature of the analysis, compulsivity was placed as the main and central theme emerging from the data. This theme demonstrated participants view that the compulsive nature of weight loss behavior is a central phenomenon in AN, and key in both the development and maintenance of weight loss behavior in AN. This was reflected in participants' feeling that this behavior characterized AN, and how they would not be “anorexic” without it.

“The compulsive nature of behavior in eating disorders is what characterizes them…if restriction/exercise weren't compulsive, anorexia would be easy to overcome because it would be a conscious choice to ‘switch off’ the thoughts and stop the behaviors. The problem is that behaviors become compulsive and so it becomes more difficult to not engage in them.”

*Participant age 21, 2 year duration of AN*

#### Theme two: Impaired control

The second theme emphasized the lack of control participants felt they had over their anorexic behaviors.

##### Sub-theme 2.1: Out of conscious control

Participants often described feeling as though their behavior was out of their control, and felt as though they were controlled or trapped by their disorder.

“Compulsion is like a tidal wave- it has so much force it just knocks you over and it is near impossible to push it out of the way and then it consumes you completely. I get scared by how much it controls me.”

*Participant age 22, 11 year duration of AN*

Participants spoke of their disorder related compulsions as being separate from themselves, and often referred to AN as a “voice” or separate entity driving them to perform these behaviors.

“The Eating Disorder (ED) has rules for food, exercise, and life in general. The compulsive part comes in when ED says you MUST restrict, over exercise, weigh a certain amount, wear a certain size, have certain body proportions, etc., and he promises that this will make you perfect, happy, and loved…. In recovery I am troubled by the compulsive nature of his voice and his rules.”

*Participant age 36, 23 year duration of AN*

A comparison to addiction was also made by some participants, describing the nature of AN as analogous to an individual feeling controlled by a substance addiction.

“It is the way it starts as a need, a compulsion to lose weight or gain control, then the habits you develop to fulfill that become ingrained and the compulsion is no longer owned by you, you become addicted and sticking to your rules is the most important thing. It also makes recovery hard because trying to ignore the compulsions you feel is like trying to get a heroin addict to turn down heroin- near on impossible.”

*Participant age 22, 11 year duration of AN*

##### Sub-theme 2.2: Go out of their way to perform compulsions

Participants described the need to overcome any obstacles in order to perform their compulsive behaviors, and being prepared to do anything to avoid being prevented from them.

“I will do anything to make sure that I am not prevented from completing compulsions—I try not to lie in my everyday life, but will often lie about things so that I can engage in compulsive behaviors.”

*Participant age 21, 2 year duration of AN*

##### Sub-theme 2.3: Desire/attempts to stop

Participants emphasized that they did not always want to perform their compulsions, and understood that they were detrimental for them, but felt unable to stop or control these behaviors.

“I feel troubled a lot and the need to do things everyday that you don't necessarily want to do is the most troubling thing—because I end up missing out on life.”

*Participant age 20, 5 year duration of AN*

Unsuccessful attempts at stopping these compulsive behaviors and at recovery in general were described, and participants also commented on their belief that any attempts to stop these behaviors would be unsuccessful.

“I can set out with the best will in the world to try and eat more or be positive, but the slightest thing can trigger the compulsion in me and then I can't hold onto that positivity. It feels as though I'm not in control of my own life—of what I want to do….”

*Participant age 21, 5 year duration of AN*

#### Theme three: Functional impairment

The third theme emphasizes the functional impairment participants describe as a result of their compulsive behaviors.

##### Sub-theme 3.1: Time consumed by compulsions

Participants described the time consuming nature of their compulsions, emphasizing that much of their time was taken up by these compulsions, or related thoughts, and the detrimental effect this had on their life.

“It consumes every waking minute of my everyday life, controlling what I can and cannot do socially and professionally, what can and cannot eat…I am most troubled by my…. inability to focus on anything else but what I have eaten/will eat.”

*Participant age 18, 4 year duration of AN*

##### Sub-theme 3.2: Interpersonal and occupational impairment

Worsening family relationships were described by participants as a result of AN. This was both due to a reduction in time spent with family due to time consuming compulsions, and worry caused to family members as a result of the participants poor health.

“Not allowing my family to see me eat has caused the most problems as it has affected dynamics in the house and my relationship with family has worsened.”

*Participant age 18, 1 year duration of AN*

Participants often commented on the all-consuming nature of AN causing problems in both their social and occupational life.

“These compulsions lead to isolation, an inability to socialize, trouble working and problems functioning at all in normal day to day life.”

*Participant age 34, 7 year duration of AN*

#### Theme four: Detrimental continuation of behavior

The fourth theme focuses on the continued engagement in compulsive behavior despite acknowledgment that it is detrimental to psychological health.

##### Sub-theme 4.1: Behavior causes psychological distress

Many of the participants describe feeling distressed or troubled by the compulsive nature of AN, and awareness that these continued behaviors are the reason behind this distress.

“Above all else, however, compulsions are what make me feel terrible about myself. They make me feel weak and a failure for not being better able to handle their demands, but also weak and a failure for having them in the first place. The need to be constantly doing something/not doing something means that I am basically in a state of dread, fear, and anxiety 24/7.”

*Participant age 22, 7 year duration of AN*

##### Sub-theme 4.2: Continued behavior despite distress

Participants described feeling unable to stop engaging in behavior despite the psychological distress caused by this behavior. Some participants also commented on the irrationality of their behavior, and felt unable to explain the reasoning behind them.

“It's terrible. Feeling like you HAVE to do something and not being able to come up with a realistic, safe, sane, rational reason for it, and knowing that it takes priority over every other aspect of your life.”

*Participant age 19, 6 year duration of AN*

#### Theme five: Escalating compulsions

Theme five describes the way in which a number of participants felt that their compulsions had increased over time. Participants often commented on a “snowball” effect of compulsions in AN, as though the compulsive behavior becomes more extreme over time as they are engaged in repeatedly, with weight loss targets/calorie rules increasing.

“The compulsion to restrict more and lose weight used to be the biggest factor in my illness: every day/week I would try to stick to an ever-decreasing calorie limit and get my weight down to a new low.”

*Participant age 36, 20 year duration of AN*

An increase in compulsions was associated with increasing weight loss, suggesting that the more weight lost, the stronger the compulsion became to engage in further weight loss behavior, and also in other compulsive behaviors.

“My experience of the compulsion in anorexia is that it grows as you lose weight. At first, restricting and exercising felt more of a choice than a compulsion but, as I lost more and more weight, restricting became more compulsive, and I would listen to the anorexia instead of trying to make my own mind up.”

*Participant age 21, 2 year duration of AN*

A spread of compulsive behavior beyond disorder related compulsions was also described by some participants; this included a compulsive attitude toward work, money, and compulsions regarding order and routine.

“I am also compulsive about academic work-I work extremely long hours on my studies, almost all hours of the day, and I get the same anxiety/feelings of failure when I don't work all the time.”

*Participant age 23, 4 year duration of AN*

#### Theme six: Reactions to prevention

Theme six describes participants' reactions to being prevented from engaging in compulsive weight loss behaviors.

##### Sub-theme 6.1: Negative feelings

The majority of participants described a very strong negative reaction to being prevented from engaging in compulsive weight loss behavior, feelings of anxiety were common.

“I feel very tense and anxious when I can't engage in a compulsive behavior. It feels very overwhelming. Like there's something bubbling up inside you and you'll just explode if you can't do the behavior to release all the tension.”

*Participant age 21, 5 year duration of AN*

Participants also often described feeling scared and unsafe when unable to engage in these behaviors.

“But if I don't do those things, I simply feel desperately unsafe, lazy, undisciplined and thoroughly bad about myself…. Hell on earth. Intolerable. Desperately unsafe. Sub-human. It simply doesn't bear thinking about. ”

*Participant age 44, 24 year duration of AN*

##### Sub-theme 6.2: Physical symptoms

A physical reaction to being unable to carry out compulsions was also described by some participants, with feelings of agitation and restlessness described, but also some more serious physiological symptoms such as tachycardia.

“When I have to increase my portion sizes I get very panicky, sometimes with physiological symptoms like tachycardia, autonomic response etc.”

*Participant age 23, 4 year duration of AN*

##### Sub-theme 6.3: Swapping one compulsion for another

Some participants described feeling an increase in the urge to perform a different weight loss compulsion to avoid the anxiety felt when a weight loss behavior is prevented.

“I can reduce the difficulty I have with exercise compulsions by restricting my food intake (and reducing the anxiety from food compulsions by increasing my amount of exercise).”

*Participant age 21, 2 year duration of AN*

#### Theme seven: Emotional triggers

The seventh theme focuses on the triggers or incentives described by participants for compulsive weight loss behaviors.

##### Sub-theme 7.1: Negative feelings

Participants often described negative feelings such as anxiety and fear of fatness as a trigger for compulsive weight loss behavior.

“To me it mostly feels as though in order to deal with life, both in terms of practicalities and emotions, the compulsion to not eat or restrict is a way for me to find a way of coping…. It is sometimes hard to see in the moment but it is certainly true that periods of greater stress and anxiety distinctly impact on my eating habits.”

*Participant age 26, 12 year duration of AN*

A feeling of being unworthy of food was also described by some participants.

“It's not quite as simple as ‘not deserving to eat,’ but I suppose that's what it boils down to, and the compulsions I feel are a huge contributing factor to that governing belief.”

*Participant age 22, 7 year duration of AN*

Participants also described feelings of low self-esteem, and its link to the development of compulsions in AN.

“I detest being this thin and judge myself accordingly, but there is an undeniable causal link between thinking very little of yourself and caring enough to get better.”

*Participant age 22, 7 year duration of AN*

##### Sub-theme 7.2: Positive feelings

Participants also described the positive feelings associated with engaging in their compulsions. This often centered on feelings of safety, and a sense achievement and success from these behaviors.

“The compulsions are frustrating at times, but also comforting and increase feelings of safety.”

*Participant age 26, 8 year duration of AN*

A feeling of being in control was also described by participants.

“I feel stronger when I control my food and exercise. I have the feeling that I control something and it makes me feel better and less stressed.”

*Participant age 25, 8 year duration of AN*

#### Theme eight: Role in recovery

Theme eight focuses on the role compulsive behavior has in recovery from AN.

##### Sub-theme 8.1: Compulsivity as a barrier to recovery

Most participants described their compulsions as the main barrier they face in recovery, suggesting that recovery was dependent on breaking these behaviors. Participants often emphasized their desire to recover, but felt unable to stop or ignore the compulsion to continue their behavior.

“I believe that freeing myself from these compulsions will ease recovery but I struggle to comprehend life without some of my tendencies.”

*Participant age 18, 1 year duration of AN*

Participants also commented on the need to intervene with these behaviors early before they become engrained, at which point recovery and breaking these behaviors becomes very difficult.

“Compulsions should be caught early before they solidify into ‘behaviors.’ Need to be challenged in order to recover/manage.”

*Participant age 21, 7.5 year duration of AN*

##### Sub-theme 8.2: Strategies to reduce compulsions

Participants described a number of ways in which they tried or were able to reduce their compulsions, although many also emphasized this was very often unsuccessful.

“I think doing things and talking to people helps reduce compulsion.”

*Participant age 18, 2 year duration of AN*

Some participants also suggested that support from others and having someone to talk to was helpful in reducing compulsions.

“Support from close family helps to some extent to reduce.”

*Participant age 39, 18 year duration of AN*

A few participants also commented on the usefulness of mindfulness techniques, particularly in reducing the obsessional thoughts associated with their compulsions.

“To reduce my sense of compulsion, I have been able to use mindfulness based techniques to recognize when such irrational thoughts come to mind.”

*Participant age 21, 7 year duration of AN*

### Parallels with criteria for SUDs

The eight main themes that emerged from the thematic analysis were further examined for parallels with the four groups of the DSM-V criteria for SUDs. Six of these main themes appeared to map onto these four groups of criteria, as shown in Table 3.

**Table 3:** **Qualitative themes mapped on to the four groups of diagnostic criteria for Substance Use Disorders**.

**Diagnostic criteria for SUDs**	**Corresponding themes**
Impaired control	Theme 2: Impaired control
	Theme 5: Escalating compulsions
Social impairment	Theme 3: Functional impairment
Risky use of substance	Theme 4: Detrimental continuation
Pharmacological criteria	Theme 5: Escalating compulsions
	Theme 6: Reactions to prevention
	Theme 7: Emotional triggers

## Discussion

To our knowledge this is the first study to employ qualitative methods to explore the subjective experience of compulsive weight loss behavior in AN, and examine the validity of suggested parallels with substance dependence. Eight main themes emerged from a thematic qualitative analysis; compulsivity as central to AN, impaired control, escalating compulsions, emotional triggers, reactions to prevention, detrimental continuation, functional impairment, and role in recovery. These themes suggest that some individuals with AN view the compulsive nature of their behavior as central to the maintenance of their disorder, and as a significant barrier to recovery. A number of these individuals also acknowledge the detrimental effect this behavior has on their psychological health, and on their ability to function effectively in everyday life, however they feel unable to control their behavior despite this. In line with suggestions of a parallel with compulsive drug-seeking behavior (Scheurink et al., 2010; Zink and Weinberger, 2010; Barbarich-Marsteller et al., 2011; Kaye et al., 2013; Godier and Park, 2014a,b; Park et al., 2014), the qualitative themes showed a number of similarities with the characteristic behaviors of substance dependence, and appear to map on to DSM-V criteria for SUDs. The parallels observed between the qualitative themes and the criteria for SUDs are shown in Table 3, and will now be discussed in reference to each of the four groups of criteria; impaired control, social impairment, risky use of substance, and pharmacological criteria.

### Impaired control

The impaired control criteria for SUDs refer to an individual experiencing cravings or a strong desire to use the substance, persistent desire/unsuccessful attempts to cut down or control substance use, alongside increasing amounts or periods of time spent taking the substance, and a great deal of time spent carrying out activities relating to substance use. Theme two of this study (impaired control), which was endorsed by 62.5% (25/40) of participants, suggested that the majority of participants in this study felt impaired control over their behavior. Participants reported feeling trapped or controlled by their disorder, and described the desire/attempts to disengage from compulsive weight loss behaviors. Furthermore, 35% (14/40) of participants also reported an escalation of compulsive behavior over time (theme three, escalating compulsions), with weight loss goals and behaviors increasing over time in a similar way to the increasing amount/time over which a substance is taken.

The lack of control described by the majority of participants over these behaviors is striking, and relevant to recent theoretical accounts of the persistence of behavior in AN (Steinglass and Walsh, 2006; Walsh, 2013). Walsh (2013) proposes that excessive habit formation may contribute to the development and maintenance of compulsive behaviors in AN. This theory posits that once weight loss behavior is repeated enough, a rewarding outcome (weight loss) may only be intermittently needed, or even no longer necessary for this behavior to continue. This makes habitual behavior highly resistant to change, which may reflect participants feeling unable to control or disengage in these behaviors in this study. A shift in balance away from goal-directed control and toward excessive habit learning has been shown in substance dependence, and other compulsive disorders such as obsessive compulsive disorder (OCD) and binge eating disorder (BED) (Gillan et al., 2011, 2014, 2015; Sjoerds et al., 2013; Voon et al., 2015). These results point to a common behavioral underpinning for the persistence of behavior across disorders of compulsivity, and may be relevant to the compulsive and persistent nature of behavior in AN (Steinglass and Walsh, 2006; Walsh, 2013).

### Social impairment

The social impairment criteria for SUDs refer to continued substance use resulting in a failure to fulfill major obligations relating to work/school and home, a reduction in or giving up of important social, occupational, or occupational activities, and social or interpersonal problems caused or exacerbated by substance use. In theme three of this study (functional impairment) 37.5% (15/40) of participants described the time consuming nature of their weight loss compulsions, and the detrimental effect this had on their family, social, and occupational lives. Weight loss behaviors appear to become the most important priority in these individuals' lives, in a similar way to drug-seeking behaviors in SUDs, to the detriment of the psychological and physical health, as well as their interpersonal and occupational functioning.

### Risky use of substance

Risky use of substance in SUDs refers to recurrent use in situations in which it is physically hazardous, and continued use despite knowledge of a physical or psychological problem relating to substance use. In theme 4 of this study (detrimental continuation), which was endorsed by 45% (18/40) of participants, psychological distress as a direct result of continued compulsive weight loss behavior was acknowledged. However, participants described feeling unable to cease these behaviors despite this. Indeed reluctance to recover is often reported in AN, and has been linked to the ego-syntonic nature of symptoms in AN, and feeling as though the benefits of having AN outweigh the negatives (Nordbø et al., 2012). Whilst speculative, it may be that in both AN and SUDs to pursuit of a rewarding outcome associated with the behavior (i.e., weight loss or drug-induced “high”) may be seen to outweigh the adverse consequences of behavior and lead to continuation of this behavior.

### Pharmalogical criteria

The pharmacological criteria for SUDs refer to tolerance to and withdrawal from the substance. Tolerance, or the need to use increasing amounts of the substance to achieve the same level of intoxication, may in some ways parallel the escalating nature of compulsions described by AN participants in theme five (escalating compulsions) in which a number of individuals with AN (35%, 14/40) reported feeling the need to increase weight loss goals/behaviors over time.

The majority of participants (72.5%, 29/40) reported a strong negative reaction to being prevented from performing compulsive behaviors, most commonly in the form of negative emotions such as anxiety and guilt, but also physical feelings such as agitation, restlessness and in one case tachycardia (theme six; negative reactions). This may be comparable to the withdrawal symptoms experienced by individuals with SUDs, although withdrawal from a physical substance is acknowledged to be much more extreme. Forty percent of participants also suggested that both the avoidance of negative emotions, such as anxiety and fear of fatness, and the pursuit of positive emotions, such as safety and control, were important in triggering compulsive weight loss behaviors (theme seven; emotional triggers). This raises the possibility that compulsive weight loss behavior in AN may be used as a maladaptive emotion-regulation strategy, used to attenuate or avoid negative emotions (Harrison et al., 2010; Wildes et al., 2010; Merwin et al., 2011). Indeed drug use has been described as means of regulating emotions (Kober and Bolling, 2014), and this may serve to further reinforce compulsive behavior in both SUDs and AN.

### Clinical implications

The parallels observed between the themes of this analysis and the criteria for SUDs support the suggestion that compulsive behavior occurs trandiagnostically across psychiatric disorders (Robbins et al., 2012), and adds face validity to suggestion of potential behavioral and neurobiological parallels between AN and substance dependence (Godier and Park, 2014a). Future research in AN may benefit from investigating the potential commonalities in the underlying mechanisms of compulsive behavior, which may open up novel avenues for treatments target these mechanisms.

Indeed, in theme eight (role in recovery) of this study, 65% (26/40) of participants suggested that the compulsive nature of behavior in AN acts as a major barrier to recovery, and participants emphasized the importance of intervening early, before weight loss behaviors “solidify” into habits. This supports further investigation of the role of maladaptive habit learning in AN, shown to be dysfunctional in substance dependence and other compulsive disorders (Gillan et al., 2011, 2014, 2015; Sjoerds et al., 2013; Voon et al., 2015). The importance of repatterning habitual behaviors has previously been emphasized regarding the treatment of AN (Park et al., 2011, 2012), and some concepts previously aimed at behavioral change and habit breaking in other disorders have been translated for use in AN. Exposure Response Therapy (ERT), which targets conditioned fear responses and conditioned reward, has been used in the treatment of addiction (Kaplan et al., 2011), and some success has been found with graded exposure to food cues in AN, reducing meal-related anxiety and increasing caloric intake post treatment (Steinglass et al., 2012, 2014).

Neuromodulatory interventions have reported efficacy in reducing compulsive behavior in substance dependence and other compulsive disorders such as OCD. Repetitive Transcranial Magnetic Stimulation (rTMS), a non-invasive brain stimulation technique, has been shown to reduce cravings and consumption in substance dependence (Barr et al., 2011), decrease compulsions and obsessions in OCD (Blom et al., 2011), and reduce anxiety and potentially the urge to exercise in AN (Van den Eynde et al., 2013). Deep Brain Stimulation (DBS) is a reversible, adjustable neurosurgical treatment that involves implanting electrodes that send electrical impulses to chosen locations in the brain (Rauch, 2003). DBS to regions of the fronto-striatal circuitry, such as the Nucleus Accumbens, has been shown to be effective in the treatment of OCD and addictions (Kuhn et al., 2007; Liu et al., 2008; Müller et al., 2009; Denys et al., 2010; Figee et al., 2013). Case reports of DBS in AN patients have also reported symptom alleviation both in the presence and absence of comorbid OCD (McLaughlin et al., 2013; Wu et al., 2013).

Novel pharmacological treatments targeting addictive or compulsive behaviors may also prove useful in the treatment of compulsivity. The use of drugs that target GABA and glutamate pathways has shown some benefit in the treatment of SUD (Clarke et al., 2004; Olive et al., 2012), binge eating (Guardia et al., 2011), and OCD (Pittenger et al., 2011), and may also prove beneficial in other disorders in which compulsive behavior develops.

### Limitations

Considering the lack of research looking at the possible parallels between the compulsive nature of behavior in AN and SUDs, qualitative data capturing the subjective experience of compulsive weight loss behaviors may be an important stepping-stone in identifying avenues for future research and treatment development. However, some important limitations should be acknowledged. Compulsive behavior was not defined prior to the prompt questions, as this concept was introduced prior to the study, and as such it is possible that there may have been discrepancies in participants understanding of this concept. Additionally, whilst the prompt questions used in this study were designed to direct responses toward the compulsive nature of behavior in AN for the purpose of the study, the wording of these prompt questions is likely to have biased participants' responses. These questions assumed participants agreement that their behavior is compulsive, and reduces the likelihood of participants discussing other potentially important symptoms. Furthermore, the deductive nature of the qualitative analysis, which was carried out with certain hypotheses in mind, is likely to have led to a selection bias in the excerpts/participants used and the themes extracted from the data. It is also true that some themes were more highly endorsed by participants than others (See Table 2). This should be considered when making a comparison to SUDs, as some parallels may be more widely relevant to AN than others. For example, whilst theme six (reactions to prevention) was endorsed by 72.5% (29/40) of participants, theme three (functional impairment) was endorsed by only 37.5% (15/40) of participants.

A more general limitation of this study was the reliance on self-report, both in terms of participants responses, and the identification of a sample of individuals with a current diagnosis of AN. This reliance on self-report may have led to inaccurate responses. This may be particularly relevant for individuals with AN, who have been shown to have low interoceptive awareness, described as the ability to differentiate between sensations and feelings (Fassino et al., 2004), and as such they may be unable to describe or report their subjective experiences accurately. The use of qualitative analysis should be interpreted cautiously, as this reliant on participants choosing to disclose certain information and personal stories, and is not directly supported by statistical analysis. The use of an online system may also be considered a limitation as this does not allow for follow up questions or clarification of responses as in interview settings. As participants were recruited as part of a larger study (Godier and Park, 2014b), sampling was not guided by the achievement of data saturation. However, the number of participants recruited is large compared to most qualitative studies, and it is felt that data saturation was achieved in the current sample, as new themes were no longer seen to be emerging by the end of transcript coding. It is however acknowledged that further participant recruitment may have resulted in the emergence of novel themes.

## Conclusions

When asked to reflect on the compulsive nature of their behavior, the majority of participants with AN described having little control over their behavior, and some reported an escalation of weight loss behavior and goals over time. Some also acknowledged the detrimental effect this behavior has on their psychological health, and on their ability to function effectively in everyday life, yet they continued to feel unable to cease this behavior. Participants' experience of compulsivity in AN appears to map on to the DSM-V criteria for SUDs, supporting increasing suggestion of parallel between these disorders, and conceptualization of compulsivity as a transdiagnostic concept. Conceptualizing compulsive behavior in this way may open up novel research and treatment avenues for AN, in which existing treatments targeting habitual and compulsive behaviors could be utilized for the treatment of AN.

## Author contributions

LG and RP both contributed the experimental design and planning of the study. LG collected the data and conducted the data analysis. Both authors contributed to the interpretation of the data, the write up and approval of the final manuscript.

## Funding

This work is funded by an MRC PhD studentship awarded to LG. RP is funded by a HEFCE Clinical Senior Lecturer award.

### Conflict of interest statement

The authors declare that the research was conducted in the absence of any commercial or financial relationships that could be construed as a potential conflict of interest.
